# The Role of Connexin 36 Gap Junctions in Retinal Ganglion Cell Death After Corneal Alkali Burns

**DOI:** 10.1167/iovs.66.12.43

**Published:** 2025-09-18

**Authors:** ChunTing Qiu, Ting Zhang, Qin Wang, Kangyi Yang, Chunghim So, Feng Pan

**Affiliations:** 1 School of Optometry, The Hong Kong Polytechnic University, Kowloon, Hong Kong; 2 Centre for Eye and Vision Research (CEVR), 17W Hong Kong Science Park, Hong Kong; 3 Hong Kong Polytechnic University Shenzhen Research Institute, Shenzhen, People's Republic of China

**Keywords:** gap junction, retinal ganglion cells (RGCs), apoptosis, alkali burns, retina

## Abstract

**Purpose:**

A corneal alkali burn can cause irreversible damage to both the cornea and retina. This study aims to investigate the role of the gap junction subunit connexin 36 (Cx36) in mediating secondary cell death and its impact on the apoptosis of retinal ganglion cells (RGCs) following ocular alkali burns, contributing to irreversible vision loss.

**Methods:**

Corneal alkali burn models were established in C57BL/6J and Cx36 knockout (KO) mice by applying 1 M sodium hydroxide to the cornea. The gap junction blocker meclofenamic acid (MFA; 200 µM) was administered via intravitreal injection immediately after the corneal alkali burn. Immunohistochemistry was used to assess RGC survival, whereas patch-clamp recording evaluated the RGC function.

**Results:**

In the mouse model, dysfunction and cell death in RGCs were observed within 6 hours following ocular alkali burns. Our results showed a time-dependent increase in RGC loss, peaking at 24 hours, with damage spreading from the peripheral to the central regions. The study revealed a significant reduction in light sensitivity and light-evoked excitatory postsynaptic currents (EPSCs) and inhibitory postsynaptic currents (IPSCs) in ON and OFF transient alpha RGCs after 6 hours of corneal alkali burns. The Cx36 knockout mice exhibited significantly increased RGC survival. The data suggests that MFA has a neuroprotective effect, preventing secondary RGC damage.

**Conclusions:**

Our findings indicate that Cx36 gap junctions mediate secondary cell death of RGCs following corneal alkali injuries and may serve as a potential target for neuroprotective therapy. The gap junction antagonist MFA, a US Food and Drug Administration (FDA)-approved drug, could prevent this secondary cell death, highlighting its potential as a therapeutic intervention.

Ocular alkali burns are medical emergencies that can rapidly cause severe damage on the ocular surface epithelium, cornea, and posterior segment.^1^ These ocular chemical burns frequently occur in the workplace, especially among young male workers, and are most often caused by sodium hydroxide and lime.^2^^,^^3^ In 2021, the incidence of ocular chemical injuries varied from 5.1 to 50 per 100,000 population annually across different countries.^4^ Alkali burns tend to be more severe than acid burns due to the ability of hydroxyl ions in alkali solutions to penetrate deeper into the eye’s surface. Without prompt and adequate treatment, patients may experience significant complications, ultimately leading to blindness.^5^

Although corneal transplants can restore clarity, nearly 60% of patients report unsatisfactory improvements in visual acuity.^6^ Accumulating evidence suggests that retinal neuron damage, particularly the apoptosis of retinal ganglion cells (RGCs), plays a crucial role in the pathophysiology of corneal alkali burns.^7^ This damage is closely linked to inflammation mediated by retinal cytokines and elevated intraocular pressure following alkali burns. The mechanism behind retinal damage following corneal alkali burns remains unclear. Current evidence suggests that the apoptosis of RGCs is not directly caused by alkali components reaching the retina as there is no change in pH value in the vitreous during the first 24 hours after an alkali burn. Instead, it is linked to the diffusion of significantly upregulated tumor necrosis factor α (TNF-α).^7^^,^^8^ Systemic administration of infliximab poses risks of significant complications, whereas topical eye drop delivery is limited by poor ocular bioavailability and the need for patient adherence.^9^ Although infliximab shows promise in managing corneal alkali burns, there are limitations and challenges for its effective application.^10^ Therefore, exploring novel neuroprotective methods is needed for advancing ocular alkali burn therapy.

Interestingly, recent studies have shown that the absence of retinal gap junctions can inhibit the activation of retinal microglia, thereby providing retinal neuroprotection.^11^^,^^12^ This suggests that retinal gap junctions may play a significant role in neuronal loss. The study examined this connection and explored new potential therapeutic pathways for mitigating vision loss caused by corneal alkali injuries.

Gap junctions (or electrical synapses), composed of two docked hemichannels called connexons, are specialized intercellular links directly connecting two cells’ cytoplasm, permitting molecules, ions, and electrical impulses to pass through a controlled gateway. Cx36 is an essential subunit of neuronal gap junctions in most retinal cell types.^13^^–^^15^ It is the predominant subunit of gap junctions in the proximal mouse retina, expressed by most RGC subtypes, including αRGCs.^16^

Secondary cell death via gap junctions is a process where damage signals are transmitted from injured cells to neighboring healthy cells, leading to the death of the latter.^12^ In this process, cell death occurs when damaged or injured cells—due to mechanical stress, toxins, or diseases—experience increased intracellular concentrations of molecules like calcium ions and reactive oxygen species, serving as damage signals. These signals are transmitted to surrounding healthy cells through gap junctions, inducing harmful effects such as increased oxidative stress and disrupted calcium homeostasis. As a result, these changes trigger a series of biochemical events within the cells, leading to a programmed cell death pathway known as apoptosis. Gap junction-mediated secondary death can result in substantial cell loss and tissue damage beyond the initial area of injury or disease. This process is significant in various pathological conditions, including neuronal degradation in brain injuries and neurodegenerative diseases, heart diseases, and retinal tissue damage.^17^^,^^18^

RGCs are the final output neurons of the vertebrate retina.^19^ In mice, there are over 40 types of RGCs, each detecting and encoding distinct aspects of visual information for transmission to the brain.^20^^–^^22^ Alpha (α) RGCs are arranged in a mosaic-like pattern across mammalian species, making them ideal for observing and evaluating retinal damage caused by corneal alkali burns. In biophysical experiments, αRGCs are studied as representatives of all RGCs. In Kcng4-YFP mice, αRGCs are labeled with a yellow fluorescent protein (YFP) to facilitate evaluation.^23^

RGCs are highly vulnerable to cellular damage and neurotoxicity.^24^ They are the primary targets in neurodegenerative and ischemic diseases.^25^ A promising therapeutic approach is the protection of RGCs by blocking gap junctions to prevent secondary cell death, which may improve the viability of RGCs following corneal alkali burns. Research indicates that Cx36 and Cx45 have opposing effects on neuroprotection under excitotoxic conditions. Genetic deletion of the gap junction subunit Cx36 increased cell survivability by approximately 50%, whereas the cell loss in Cx45 knockout mouse retinas was similar to that in wild type (WT) mice.^12^

Notably, the US Food and Drug Administration (FDA)-approved gap junction antagonist meclofenamic acid (MFA), which is currently used to treat inflammatory conditions, has demonstrated protective effects against RGC damage in experimental glaucoma.^12^ Our findings reveal that Cx36-mediated secondary cell death significantly contributes to the apoptotic degeneration of RGCs following alkali-induced ocular trauma.

## Methods

### Ethics Approval

All animal procedures were approved by the Animal Subjects Ethics Sub-Committee of the Hong Kong Polytechnic University (Approval No. 23-24/937-SO-R-GRF). All experiments complied with the ARVO Statement for the Use of Animals in Ophthalmic and Vision Research.

### Animal and Cornea Alkali Burn Mice Model

Adult WT mice of either sex (postnatal days 16–56) C57BL/6J (RRID: IMSR_JAX:000664), weighing (15–20 g), were used in the study (*n* = 93, 57 male and 36 female mice). Homozygous Cx36KO mice (Cx36^−/−^) (RRID: MGI: 3,810,172), first generated in the laboratory of David Paul, Harvard Medical School (Cambridge, MA, USA), were a kind gift from Samuel M. Wu, Baylor College of Medicine (*n* = 16, 11 male and 5 female mice, 6–8 weeks old, weight 15–25 g). All animals were maintained in a 12-hour light/12-hour dark cycle. For consistency, all mouse retinas were collected at approximately 10 AM after overnight dark adaptation.

An ocular alkali burn was induced on the right eye (the left eye served as the control) of the mouse using the following method. The mice were anesthetized via intraperitoneal injection of ketamine (80 mg/kg) and xylazine (10 mg/kg), with lidocaine (20 mg/mL) applied locally to the eyelids and surrounding tissues prior to enucleation. A toe pinch test confirmed deep anesthesia. A 2-mm-diameter filter paper was soaked in 1 mol/L sodium hydroxide solution for 10 seconds and then placed on the mouse cornea center. Subsequently, 2 µL of 1 mol/L sodium hydroxide solution was applied to the filter paper for 30 seconds. After removing the filter paper, the cornea was promptly irrigated with sterile saline for 20 seconds. The anesthetized mouse was then placed laterally on a heating pad for up to 24 hours.

Then, the eyes were enucleated. The deep anesthetized mice were killed by cervical dislocation immediately after enucleation.

### Retina Preparation for Recording and Intracellular Dye Injections

Eyes were removed under dim red illumination and hemisected anterior to the ora serrata. The anterior optical structures and the vitreous humor were removed, and the resultant retina eyecup with the sclera attached, either whole or in sections, was placed in a superfusion chamber. Retinas were dissected into four equal quadrants for patch recordings and attached to a modified translucent Millicell filter ring (Millipore, Bedford, MA, USA). The flattened retinas were superfused with oxygenated mammalian Ringer's solution, pH7.4, at 32°C.

For intracellular injection, sharp electrodes with a resistance of approximately 100 MΩ were used. The pipette tips were filled with 4% Neurobiotin (Vector Laboratories, Burlingame, CA, USA) and 0.5% Lucifer Yellow-CH (Molecular Probes, Eugene, OR, USA) in double-distilled water, and then backfilled with 3 M KCl. The cells were injected with a biphasic current of +1.0 nA at 3 hertz (Hz) for 15 seconds, followed by a 10-minute diffusion period. As to cytochrome C (CytC) intracellular injections to induce secondary cell death, neurons were visualized and injected with CytC (10 mg/mL, 1 millimolar [mM]) and 4% Neurobiotin in 0.1 M Tris buffer. Substances were injected iontophoretically for 15 to 20 minutes using a sinusoidal current (3 Hz, 1 nA).

Extracellular recordings were obtained from RGCs in all retinal quadrants, as previous described.^12^

Whole-cell recordings were performed by using an Axopatch 700B amplifier connected to Digidata 1550B interface and pCLAMP 10 software (Molecular Devices). Cells were visualized with near infrared light (>775 nm) at × 40 magnification with a Nuvicon tube camera (Dage-MTI, Michigan City, IN, USA) and differential interference optics on a fixed-stage microscope (Eclipse FN1; Nikon, Tokyo, Japan). Retina were superfused at a rate of 1 to 1.5 mL per minute with a Ringer solution composed of (mM): 120 NaCl, 2.5 KCl, 25 NaHCO_3_, 0.8 NaHPO_4_, 0.1 NaH_2_PO_4_, 1 MgCl_2_, 2 CaCl_2_, and 5 D-glucose. The bath solution was continuously bubbled with 95% O_2_ 5% CO_2_ at 32°C.

Electrodes were pulled to 5 to 7 MΩ resistance, with internal solutions consisting of (mM): 120 potassium gluconate, 12 KCl, 1 MgCl_2_, 5 EGTA, 0.5 CaCl_2_, 0.2GTP, and 10 HEPES (pH adjusted to 7.4 with KOH). This internal solution was used in experiments where spiking was not blocked. Spike trains were recorded digitally at a sampling rate of 20 kilohertz (kHz) with Axoscope software, with sorting by using Off-line Sorter (Plexon, Dallas, TX, USA) and NeuroExplorer (Nex Technologies, Littleton, MA, USA) software.

A green (525 nm) light-emitting diode delivered uniform full-field visual stimulation on the retina's surface.

Light intensity–response profiles for individual cells were generated by calculating spike counts or current amplitudes in 500 ms bins before, during and after the presentation of a stimulus of 500 ms duration with intensities varied over 5 log units. The number of light-evoked ON and OFF spikes of RGCs or current amplitudes were calculated by subtracting the background spike or current activity from those evoked by the light stimulus onset and offset, respectively. Cells were classified as sustained or transient based on spike frequency parameters as described by Della Santina.^26^

The sensitivity curves were achieved by plotting light-evoked responses against light intensities with Origin software (Origin Lab, Northampton, MA, USA). For comparison, the response of each cell was normalized by dividing its maximal response. The data points were fitted by the Michaelis-Menten equation:

$$\begin{array}{c} R=\mathrm{Rmax}*I/\left(I50+I\right) \end{array}$$

where R is the light-evoked response, I is the light intensity, I50 is the light intensity that induces a half-maximal response, and Rmax is the maximum response. The sensitivity threshold was determined as the light intensity that can induce 5% of maximum response. Averaged response data were then normalized and plotted against the intensity of the light stimuli.^27^^,^^28^

### Intravitreal Injection

Intravitreal injections in mice were performed 3 mm posterior to the limbus. A 15-degree knife was used to penetrate the sclera, and MFA was administered via intravitreal injection at a dosage of 2 µL with a concentration of 200 µM, using a Hamilton needle. Additionally, a HEPES solution was administered via intravitreal injection at a dosage of 1.5 µL with a concentration of 0.1 M as a vehicle control.

### Immunohistochemistry and TUNEL Labeling

For the immunohistochemistry experiment, the retinas were extracted from the mice and fixed in 4% paraformaldehyde for 20 minutes. Then, the whole-mount retinas were labeled with a mouse Anti-Brn-3a antibody (RRID: AB_626765, Santa Cruz Biotechnology, Dallas, TX, USA, SC8429) or goat anti-ChAT antibody (1:100, RRID:AB_2079751. Millipore Cat# AB144P).

Following the alkali burns, the whole-mount retinas were prepared. Apoptotic cells were detected using TUNEL (Tdt-mediated dUTP-X-nick end labeling) with the In Situ Cell Death Detection Kit, TMR red (Roche, Basel, Switzerland). Before slide mounting, 4′,6-diamidino-2-phenylindole (DAPI; Sigma-Aldrich, US, D9542) was added.

To conduct a quantitative analysis of TUNEL-positive cells, each whole-mount retina was divided into three regions: far-peripheral, mid-peripheral, and central areas. From each region, 4 images were selected, yielding in a total of 12 images per mouse for retinal cell counting. The images were processed and analyzed by using ImageJ software (ImageJ, an open source developed by National Institutes of Health, Bethesda, MD, USA, 1.54 g, RRID: nif-0000-30467). All data are presented as mean ± SEM. Comparisons between the two groups were analyzed using an unpaired *t*-test, whereas comparisons among the three groups across different regions were analyzed using a one-way analysis of variance followed by Tukey's multiple comparison test. Statistical analysis was performed using commercial statistical software (Prism 9.5.1 [RRID: SCR_002798]; GraphPad, Inc., La Jolla, CA, USA). A *P* value of less than 0.05 was considered statistically significant.

## Results

### Corneal Alkali Burns Result in the Death of Retinal Ganglion Cells

**Figure 1.**
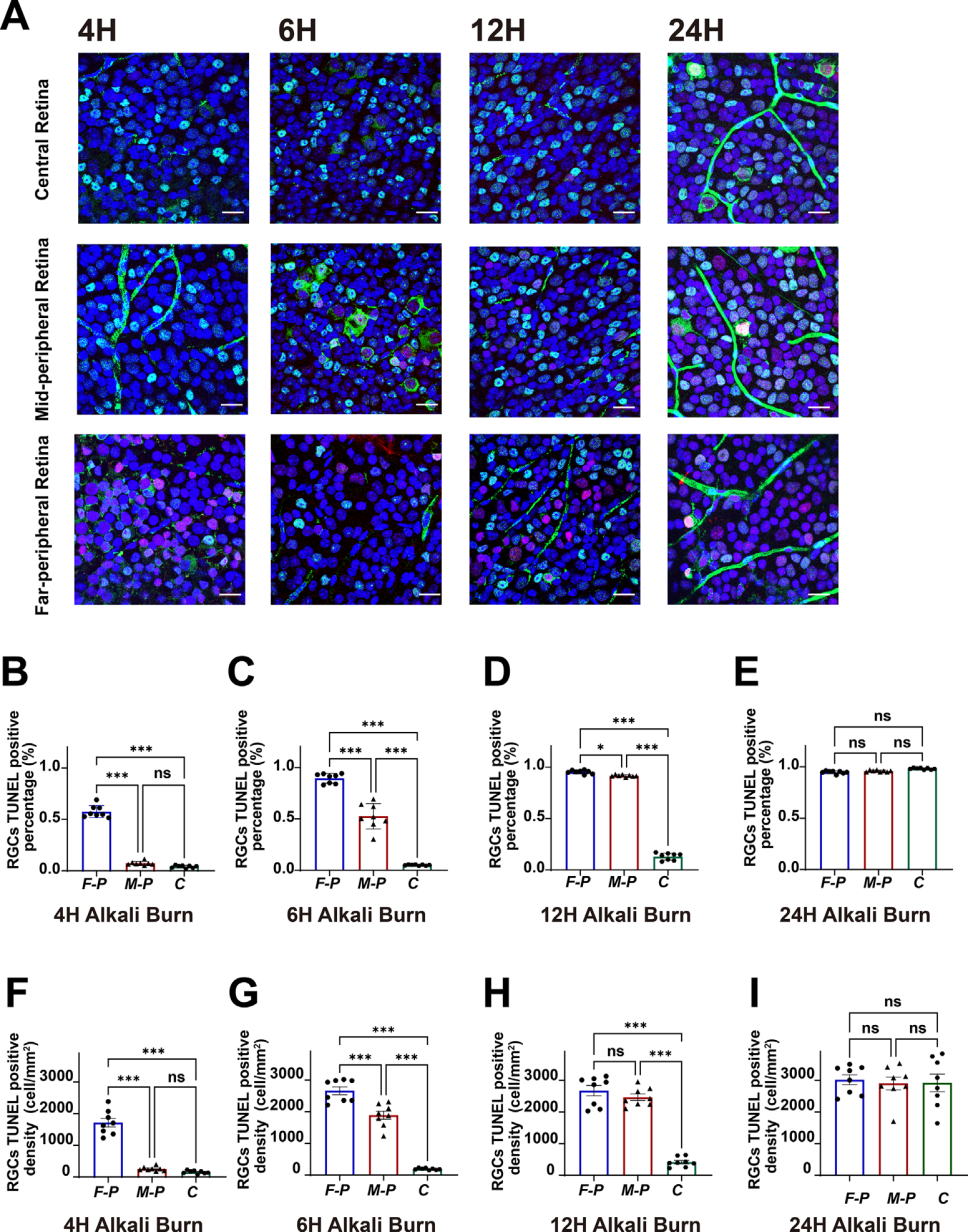
**The apoptosis of retinal ganglion cells resulting from corneal alkali burns**. (**A**) Representative confocal images of retinal whole-mounts from C57BL/6J mice taken at 4, 6, 12, and 24 hours after corneal alkali burn (*green* = Brn3a; *red* = TUNEL; and *blue* = DAPI). The percentage of TUNEL-positive nuclei in the RGC layer across three retinal regions—far-peripheral (F-P), mid-peripheral (M-P), and central (C)—at 4 hours (**B**), 6 hours (**C**), 12 hours (**D**), and 24 hours (**E**) post-alkali burn. The density of TUNEL/Brn3a double-positive cells was assessed in three retinal regions (F-P, M-P, and C)—at 4 hours (**F**), 6 hours (**G**), 12 hours (**H**), and 24 hours (**I**) following alkali burn. Statistical analysis was performed using 1-way ANOVA followed by Tukey's multiple comparison test; ns, not significant; *, *P* < 0.05; **, *P* < 0.01; ***, *P* < 0.001. *Scale bars* represent 20 µm.

To elucidate the effects of alkali-induced corneal injury on retinal neurons, we examined the onset and progression of RGC apoptosis within the initial 24 hours post-injury. We used Brn3a as a marker for RGCs, and the TUNEL assay to identify apoptotic cells, comparing cell quantities between control eyes and those subjected to corneal alkali burns at 4, 6, 12, and 24 hours post-injury. The results revealed a significant temporal and spatial progression of TUNEL-positive RGCs in the RGC layer following the corneal alkali burn (Fig. 1A). The apoptotic process began in the far-peripheral retina and gradually extended to the mid-peripheral and central regions over time.

At 4 hours post-injury, TUNEL-positive reactions were primarily observed in the retinal periphery (see Fig. 1A). Quantitative analysis of TUNEL-positive RGC counts (Fig. 1B) showed a significant increase in apoptotic cells in the far-peripheral area (0.58 ± 0.03, mean ± SEM, *n* = 8) compared with both the mid-peripheral (0.07 ± 0.01, *n* = 8) and central areas (0.04 ± 0.01, *n* = 8), which exhibited similar, lower levels of apoptosis. Consistently, the density of double-labeled TUNEL-positive and Brn3a-positive cells was significantly higher in the far-peripheral area (1714 ± 137 cells/mm², *n* = 8) compared to the mid-peripheral area (253 ± 24 cells/mm², *n* = 8), and lowest in the central area (154 ± 18 cells/mm², *n* = 8; Fig. 1F).

By 6 hours post-injury, TUNEL-positive reactions began to appear in the equatorial region, although they remained predominantly in the retinal periphery (Fig. 1A). Quantitative assessment (see Fig. 1C) showed a significant increase (*P* < 0.05) in TUNEL-positive RGCs in the far-peripheral area (0.90 ± 0.02, *n* = 8), whereas the mid-peripheral (0.53 ± 0.04, *n* = 8) and central areas (0.05 ± 0.01, *n* = 8) maintained lower levels. Correspondingly, the density of double-labeled TUNEL-positive and Brn3a-positive cells was also significantly highest in the far-peripheral area (2626 ± 124 cells/mm², *n* = 8), intermediate in the mid-peripheral area (1889 ± 128 cells/mm², *n* = 8), and substantially lower in the central area (189 ± 11 cells/mm², *n* = 8; see Fig. 1G).

At 12 hours post-injury, TUNEL-positive reactions were observed in the central region, but most apoptotic activity continued to be concentrated in the far-peripheral and mid-peripheral areas (see Fig. 1A). Quantitative analysis (Fig. 1D) revealed significantly higher levels in TUNEL-positive RGCs in both the far-peripheral (0.96 ± 0.01, *n* = 8) and mid-peripheral (0.92 ± 0.01, *n* = 8) areas, whereas the level of RGC apoptosis remained significantly lower in the central region (0.13 ± 0.02, *n* = 8). Similarly, the density of TUNEL/Brn3a double-positive cells was high in both the mid-peripheral (2674 ± 165 cells/mm²) and far-peripheral regions (2468 ± 112 cells/mm²), but was significantly lower in the central area (416 ±111 cells/mm²; Fig. 1H).

Twenty-four hours after injury, TUNEL-positive reactions were observed throughout the entire retina (Fig. 1A). Quantitative analysis (see Fig. 1E) showed a significant increase in TUNEL-positive RGCs across all retinal regions, with values recorded as follows: the far-peripheral area at 0.95 ± 0.01 (*n* = 8), the mid-peripheral at 0.96 ± 0.01 (*n* = 8), and the central area at 0.98 ± 0.01 (*n* = 8). These consistently high levels of apoptosis indicate widespread cell death. The density of TUNEL/Brn3a double-positive cells showed a central-to-peripheral gradient, with values in the central areas (2926 ± 277 cells/mm²), followed by the mid-peripheral (2906 ± 199 cells/mm²) and the far-peripheral regions (3035 ± 281 cells/mm²; Fig. 1I).

These findings reveal a distinct spatiotemporal pattern of RGC apoptosis following a corneal alkali burn, starting in the far-peripheral retina and progressively moving toward the central retina over 24 hours. This pattern suggests a potential wave of retinal damage spreading from the periphery inward, possibly due to the diffusion of inflammatory mediators or other injury-related signals from the cornea to the retina.

### Blocking Cx36-Related Gap Junctions May Reduce Cell Death From Corneal Alkali Burns

**Figure 2.**
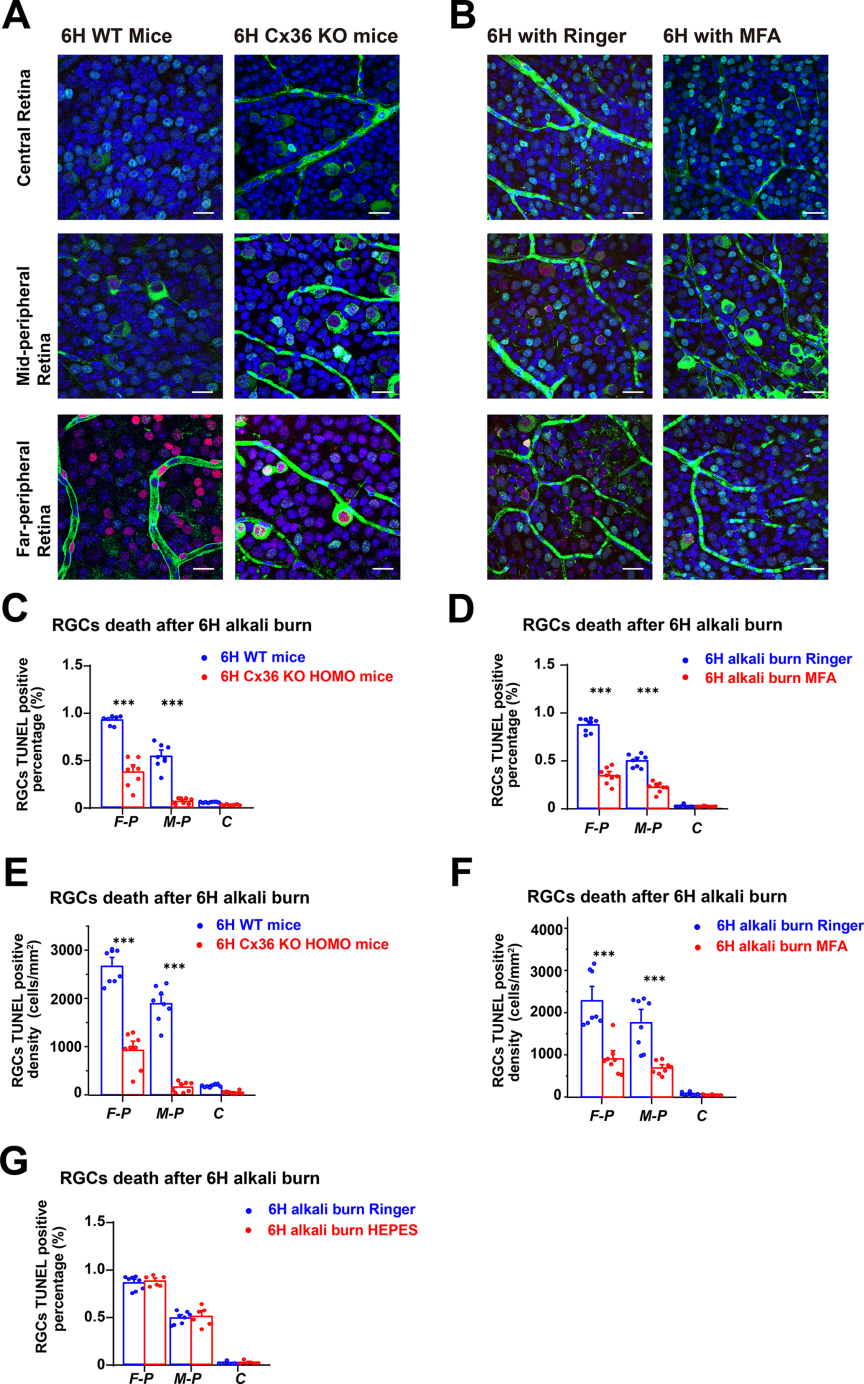
**Retinal damage following** **6-hour corneal alkali burns.** (**A**) Representative confocal images of retinal whole-mounts from wild-type (WT) mice and Cx36 knockout (KO) HOMO mice, 6 hours post-corneal alkali burn (*green* = Brn3a; *red* = TUNEL; and *blue* = DAPI and *green* = Brn3a; *red* = TUNEL; *blue* = DAPI, respectively). (**B**) Representative confocal images of retinal whole-mounts from WT mice treated with Ringer’s solution and MFA after 6 hours post-corneal alkali burns. (**C**) Percentage of TUNEL-positive nuclei in RGCs across 3 regions in WT and Cx36 KO HOMO mice after 6 hours post-corneal alkali burns (F-P = far-peripheral, M-P = mid-peripheral, and C = central retinas). (**D**) Percentage of TUNEL-positive nuclei in RGCs across three regions in WT mice treated with Ringer’s solution and MFA after 6 hours post-corneal alkali burns. (**E**) Density of TUNEL-positive nuclei in RGCs across 3 regions in WT and Cx36 KO HOMO mice after 6 hours post-corneal alkali burns. (**F**) Density of TUNEL-positive nuclei in RGCs across 3 regions in WT mice treated with Ringer’s solution and MFA after 6 hours post-corneal alkali burns. (**G**) Percentage of TUNEL-positive nuclei in RGCs across three regions in WT mice treated with intraocular injection of 1.5 µL of 0.1 M HEPES buffer and Ringer’s solution 6 hours post-corneal alkali burns. Statistical analysis = unpaired *t*-test; *, *P* < 0.05; **, *P* < 0.01; ***, *P* < 0.001. *Scale bars* represent 20 µm.

To investigate the potential protective effect of a Cx36-related gap junction antagonist on RGC apoptosis following corneal alkali burns, we first established a 6-hour corneal alkali burn model in both WT and Cx36 knockout (KO) mice (Fig. 2A). This allowed us to observe αRGC apoptosis in the far-peripheral, mid-peripheral, and central retinal areas. The primary difference between WT and Cx36 KO mice was observed in the far-peripheral and mid-peripheral retinal areas. As illustrated in Figure 2C, compared to the far-peripheral (0.90 ± 0.02, *n* = 8) and mid-peripheral (0.53 ± 0.09, *n* = 8) retina of 6 hours in the WT group, there was a notable reduction in TUNEL-positive reactions in the far-peripheral area (0.36 ± 0.06, *n* = 8) and the mid-peripheral area (0.06 ± 0.01, *n* = 8) of the 6-hour Cx36 KO homozygous mice group. The density of TUNEL/Brn3a double-positive cells in this region was significantly lower in Cx36 KO homozygous mice (the far-peripheral = 917 ± 146 cells/mm² and the mid-peripheral = 159 ± 37 cells/mm²) compared with WT mice (the far-peripheral = 2626 ± 124 cells/mm² and the mid-peripheral = 1889 ± 128 cells/mm²; Fig. 2E). Statistical analysis confirmed a significant decrease in TUNEL-positive reactions for Cx36 KO homozygous mice in this area.

To further investigate the therapeutic potential of Cx36-related gap junction protection, we administered MFA, a known gap junction blocker, via intravitreal injection as our experimental treatment, using Ringer’s solution as the control (Fig. 2B). Consistent with our genetic Cx36 KO study, we quantified the apoptosis of RGCs in the far-peripheral, mid-peripheral, and central retinal areas (Fig. 2D). Similar to the results observed in Cx36 KO mice, a significant decrease in RGC apoptosis was noted in the far-peripheral (0.34 ± 0.03, *n* = 8) and the mid-peripheral retina following MFA application (0.22 ± 0.02, *n* = 8). In contrast, without MFA treatment (using Ringer solution as the control), a significantly higher number of TUNEL-positive reactions were observed in the far-peripheral (0.87 ± 0.03, *n* = 8) and the mid-peripheral retina (0.51 ± 0.03, *n* = 8). Consistently, the density of TUNEL/Brn3a double-positive cells in this area was significantly lower in MFA-treated mice (the far-peripheral = 903 ± 130 cells/mm² and the mid-peripheral = 688 ± 54 cells/mm²) compared to the Ringer-treated control group (the far-peripheral = 2290 ± 230 cells/mm² and the mid-peripheral = 1633 ± 205 cells/mm²; Fig. 2F).

These findings suggest that the genetic knockout of Cx36 and pharmacological blocking with MFA offer a protective effect against RGC apoptosis following a 6-hour corneal alkali burn, particularly in the mid-peripheral retina.

To investigate whether alkali could directly affect retinal neurons, we administered an intraocular injection of 1.5 µL of 0.1 M HEPES buffer into the vitreous cavity to observe the extent of RGC apoptosis. Ringer’s solution was used as a control. Six hours after inducing an alkali burn on the cornea, we observed no significant differences in the far-peripheral, mid-peripheral, and central regions between the HEPES group (far-peripheral = 0.89 ± 0.02, mid-peripheral = 0.51 ± 0.04, and the central regions = 0.01 ± 0.01, *n* = 6) and the Ringer’s solution control group (far-peripheral = 0.87 ± 0.03, mid-peripheral = 0.49 ± 0.02, and the central regions = 0.01 ± 0.01, *n* = 8; Fig. 2G).

### Morphology of ON and OFF αRGCs Unaffected by Six-Hour Corneal Alkali Burn

**Figure 3.**
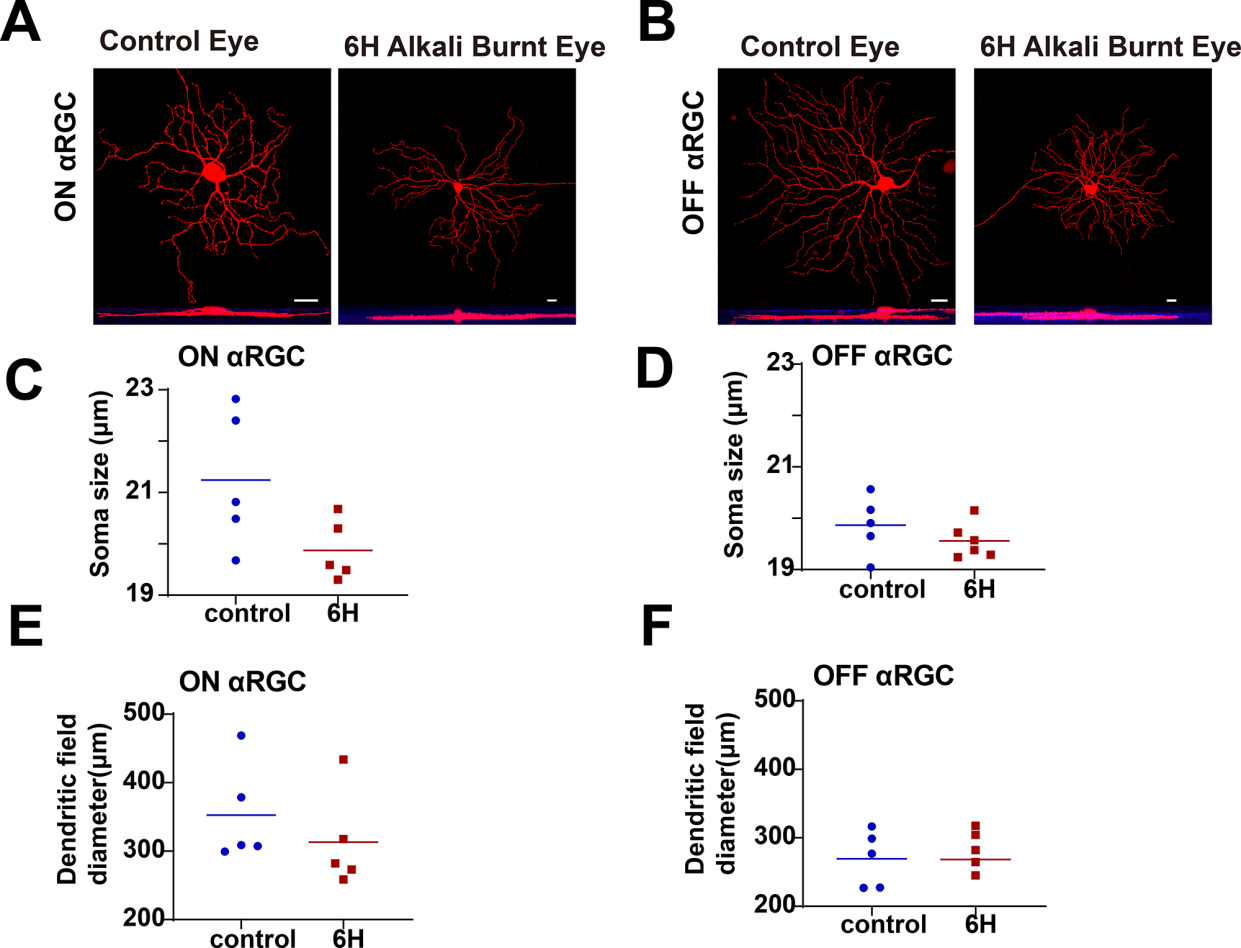
**ON and OFF αRGCs in the retinas of** **WT** **mice before and after** **6-hour corneal alkali burns.** (**A, B**) Representative images of ON and OFF αRGCs in the mouse retina. ON and OFF αRGCs were labeled with anti-ChAT antibody (*blue*). (**C, D**) Soma diameter size of ON and OFF αRGCs showed no statistical difference between the WT mouse retinas and those subjected to 6-hour corneal alkali burns. (**E, F**) Similarly, the dendritic field diameter of ON and OFF αRGCs showed no statistical difference between the WT mouse retinas and those after 6-hour corneal alkali burns. *Scale bars* represent 20 µm.

To evaluate the effects of 6-hour corneal alkali burns on the morphology of ON and OFF αRGCs, we examined 2 key aspects of neuronal structure: soma diameter and dendritic field diameter (Figs. 3A, 3B). Our analysis revealed no significant differences in the soma size of ON αRGCs and OFF αRGCs both before (ON αRGCs = 21.24 ± 0.59 µm and OFF αRGCs = 19.87 ± 0.26 µm, *n* = 5) and after the alkali burn (ON αRGCs = 19.87 ± 0.26 µm and OFF αRGCs = 19.56 ± 0.14 µm, *n* = 5; Figs. 3C, 3D). Similarly, the dendritic field diameters showed no significant changes before (ON αRGCs = 348.80 ± 26.70 µm and OFF αRGCs = 269.30 ± 18.33 µm, *n* = 5) and after the burn (ON αRGCs = 313.10 ± 31.75 and OFF αRGCs = 268.50 ± 17.84 µm, *n* = 6; Figs. 3E, 3F).

### Corneal Alkali Burns Reduce Light-Evoked Spike Responses in ON and OFF Transient αRGCs

**Figure 4.**
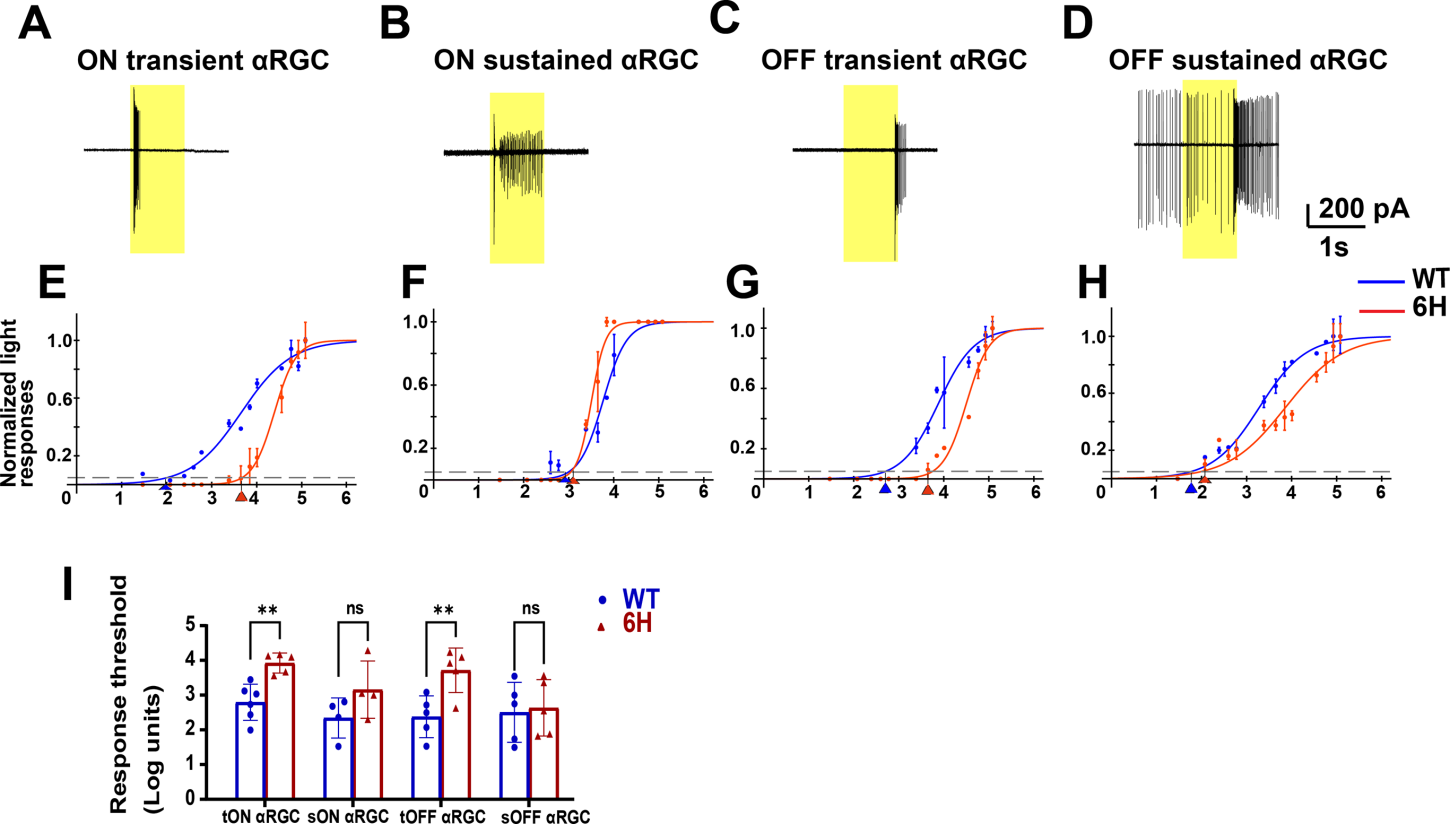
**Light responses of αRGCs in** **WT** **and** **6-hour alkali burn groups.** (**A–D**) Example spike responses of ON transient, ON sustained, OFF transient, and OFF sustained αRGCs to full-field 525 nm light stimuli. (**E–H**) Fitted normalized response-intensity curves for the 4 αRGC subtypes in the control group (*blue*) and the 6-hour alkali burn group (*red*). (**I**) Light response thresholds for the 4 αRGC types in WT and 6-hour alkali burn mice. Average thresholds are defined as the light intensity eliciting 5% of the maximum spike response. ns, not significant; *P* > 0.05; *: *P* 0.05 > *P* > 0.01; **: *P* 0.01 > *P* > 0.001; ***: *P* < 0.001.

The light sensitivity of RGCs was evaluated to determine the effects of a 6-hour corneal alkali burn. The αRGCs were classified into 4 distinct subtypes based on their responses to 525 nm full-field light: ON transient, ON sustained, OFF transient, and OFF sustained (Figs. 4A–D).

Figures 4E and 4G highlighted a notable rightward shift in the light-evoked spike response curves for ON transient and OFF transient RGCs, respectively, in the 6-hour corneal alkali burn retina compared to the WT mouse retina. Specifically, the ON transient RGCs in Figure 4E shifted from 1.0 × 10² in WT to 4.7 × 10³ Rh*/rod/second after the burn, and the OFF transient RGCs in Figure 4G shifted from 5.0 × 10² in WT to 5.0 × 10³ Rh*/rod/second. This shift indicated a reduction in light sensitivity following corneal injury. However, there were no significant differences observed in the ON sustained RGCs in Figure 4F (from 6.3 × 10² in WT to 1.3 × 10³ Rh*/rod/second after the burn) and OFF sustained RGCs in Figure 4H (from 0.5 × 10² in WT to 0.6 × 10² Rh*/rod/second after the burn).

Statistical analysis indicated that the spike response thresholds for ON transient αRGCs and OFF transient αRGCs in the 6-hour corneal alkali burn retina (ON transient αRGCs = 3.92 ± 0.13, *n* = 5 and OFF transient αRGCs = 3.71 ± 0.29, *n* = 5) were significantly higher (0.01 > *P* > 0.001) than those in the WT mouse retina (ON transient αRGCs = 2.79 ± 0.21, *n* = 6 and OFF transient αRGCs = 2.38 ± 0.27, *n* = 5), approximately one log unit greater. These findings are visually represented in the bar graph in Figure 4I.

Interestingly, our data showed no significant difference in light response thresholds between the WT mouse retina (ON sustained αRGCs = 2.34 ± 0.29, *n* = 4 and OFF sustained αRGCs = 2.50 ± 0.39, *n* = 5) and the 6-hour alkali-burnt mouse retina for the 2 sustained αRGC subtypes (ON sustained = 3.16 ± 0.41, *n* = 4 and OFF sustained = 2.63 ± 0.36, *n* = 5), as illustrated in Figures 4F, 4H, and 4I. This differential effect on transient versus sustained αRGCs may suggest a selective vulnerability of certain RGC subtypes to corneal alkali burn-induced changes in retinal function.

### Substantial Reduction in Light-Evoked Excitatory Postsynaptic Currents and Inhibitory Postsynaptic Currents in ON and OFF αRGCs After Six-Hour Corneal Alkali Burn

Using whole-cell recording techniques, we aimed to determine whether corneal alkali burns affect the strength of light-evoked excitatory postsynaptic currents (EPSCs) and inhibitory postsynaptic currents (IPSCs) in RGCs.

**Figure 5.**
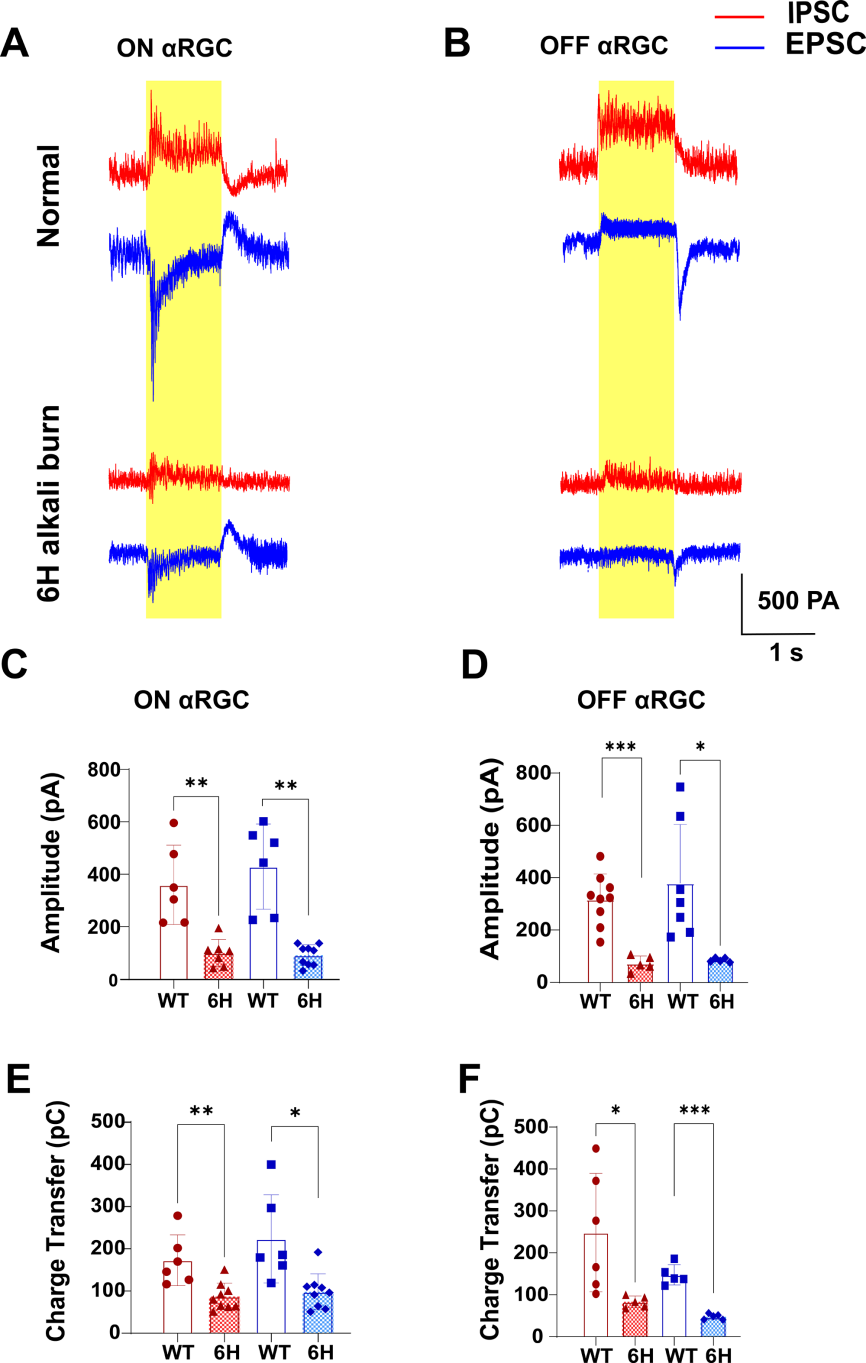
**Light-evoked EPECs and IPSCs in ON and OFF αRGCs of** **WT** **and** **6-hour alkali burn groups.** EPSCs and IPSCs were recorded at holding potentials of −68 mV and +20 mV, respectively, with EPSCs shown in *blue* and IPSCs in *red*. (**A, B**) Images of light-evoked EPSCs and IPSCs in ON and OFF αRGCs from WT and 6-hour alkali burn mice. (**C, D**) Average peak amplitudes of light-evoked EPSCs and IPSCs in ON and OFF αRGCs. (**E, F**) Average total charge transferred by light-evoked EPSCs and IPSCs in ON and OFF αRGCs. ns, not significant *P* > 0.05; *: *P* 0.05 > *P* > 0.01; **: *P* 0.01 > *P* > 0.001; ***: *P* < 0.001; ****: *P* < 0.0001.

In WT mice, ON αRGCs exhibited large, sustained inward currents in EPSCs (626.5 ± 24.7 pA) under full-field light stimulation. However, after the 6-hour corneal alkali burn, ON αRGCs displayed smaller, transient inward currents (in 129.2 ± 8.37 pA). Similarly, in IPSCs recordings, ON αRGCs showed sustained outward currents (465 ± 16.49 pA), which changed to transient outward currents (195.10 ± 6.20 pA) in the corneal alkali burn group; Fig. 5A). This indicated a significant reduction in the strength of light-evoked EPSCs and IPSCs in ON αRGCs post-injury. Compared to the WT group (EPSCs = 429.2 ± 66.26 pA, *n* = 6 and IPSCs = 359.80 ± 61.62 pA, *n* = 6), the alkali burn group (EPSCs = 91.84 ± 13.18 pA, *n* = 9 and IPSCs = 100.30 ± 19.44 pA, *n* = 7) showed reduced average peak amplitudes for both EPSCs and IPSCs (Fig. 5C; Welch's *t*-test). During the 1-second light stimulation, charge transfer for both EPSCs and IPSCs was significantly diminished in the burn group (EPSCs = 98.52 ± 14.16 pC, *n* = 9 and IPSCs = 87.22 ± 10.57 pC, *n* = 9) compared to the WT group (EPSCs = 223.90 ± 42.69 pC, *n* = 6 and IPSCs = 173.50 ± 24.60 pC, *n* = 6; Fig. 5E, unpaired *t*-test for IPSCs and the Welch's *t*-test for EPSCs).

We also investigated the impact on OFF αRGCs by recording their light-evoked EPSCs and IPSCs. Figure 5B presents representative EPSCs and IPSCs from OFF αRGCs in each group, recorded at holding potentials of −68 mV and +20 mV, respectively. Similar to ON αRGCs, the OFF αRGCs exhibited a shift from large, sustained inward currents to smaller, transient currents after the burn (from 558.9 ± 38.59 pA to 93.7 ± 4.00 pA). A similar trend was observed in the outward IPSC recordings (from 318.8 ± 25.6 pA to 107.1 ± 0.90 pA). Compared to the WT group (EPSCs = 379.30 ± 84.50 pA, *n* = 7 and IPSCs = 317.00 ± 32.53 pA, *n* = 9), OFF αRGCs in the burn group (EPSCs = 85.70 ± 3.55 pA, *n* = 5 and IPSCs = 73.20 ± 12.87 pA, *n* = 5) showed reduced average peak amplitudes for both EPSCs and IPSCs (Fig. 5D, Welch's *t*-test). The difference in charge transfer between the groups was statistically significant for both EPSCs (WT = 147.60 ± 10.83 pC, *n* = 5 and 6-hour = 47.77 ± 3.07 pC, *n* = 5) and IPSCs (WT = 248.70 ± 57.63 pC, *n* = 6 and 6-hour = 3.63 ± 6.08 pC, *n* = 5; Fig. 5F, Welch's *t*-test).

### MFA Treatment in Six-Hour Alkali-Burned Eyes Reduces Light Intensity Loss Without Altering Light Sensitivities of ON and OFF Transient αRGC

We investigated the potential of the gap junction antagonist MFA to alleviate the reduced light response threshold in transient RGCs following a corneal alkali burn. Mice subjected to the burn were divided into two groups: the experimental group received intravitreal injections of MFA, whereas the control group received Ringer’s solution for comparison.

**Figure 6.**
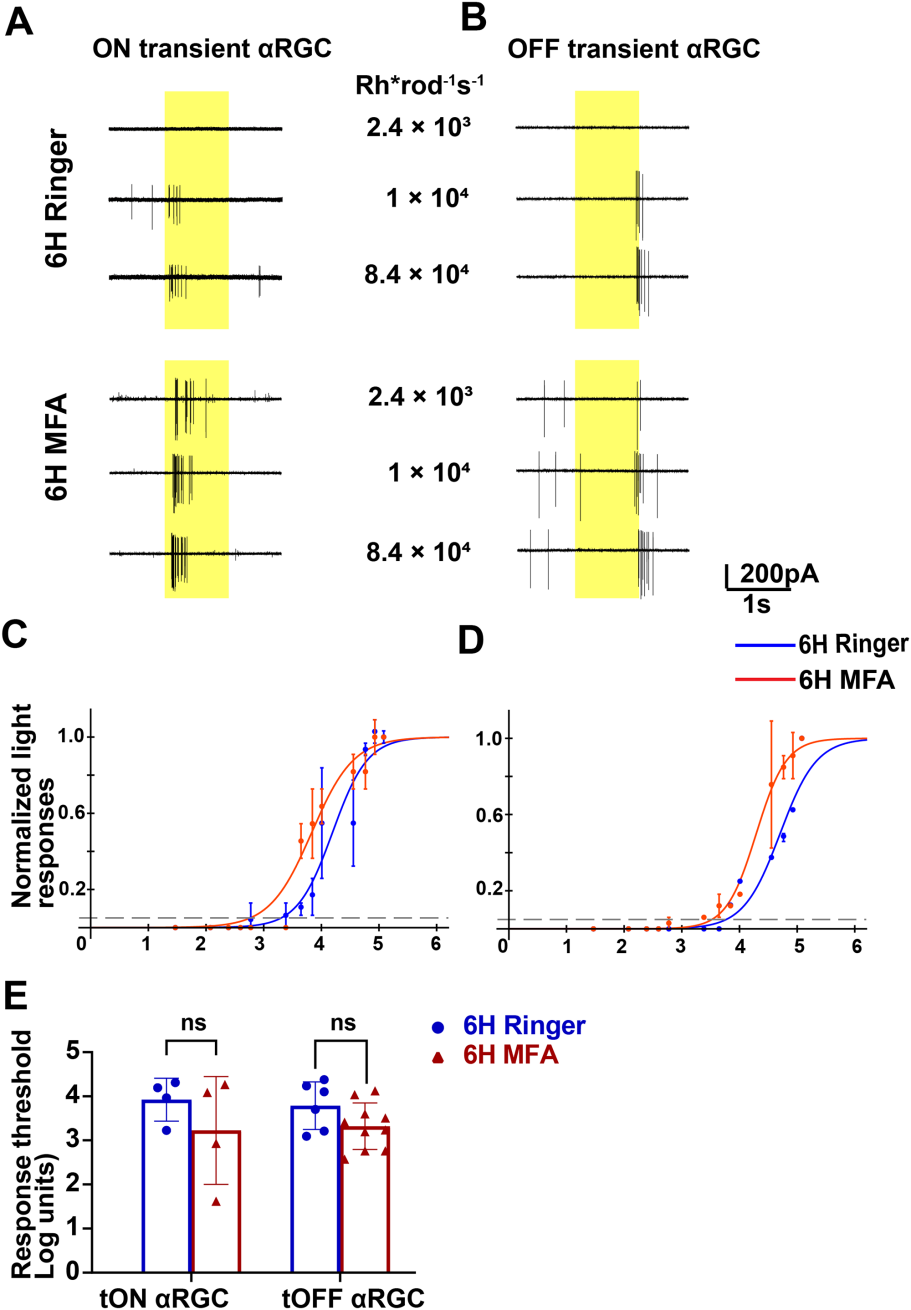
**Light responses of ON and OFF transient αRGCs in** **6-hour alkali burn of** **the** **Ringer’s solution** **and** **the** **MFA treated groups.** (**A, B**) Example spike responses of ON and OFF transient αRGCs to full-field 525 nm light stimuli at varying intensities. (**C, D**) Normalized light response-intensity curves for the two transient αRGC subtypes in the 6-hour alkali burn in Ringer’s solution (*blue*) and the MFA-treated (*red*) groups. (**E**) Light response thresholds for the 2 αRGC subtypes in the 6-hour alkali burn Ringer’s solution and the MFA treated groups. Average thresholds are defined as the light intensity eliciting 5% of the maximum spike response. ns, not significant, *P* > 0.05; *: *P* 0.05 > *P* > 0.01; **: *P* 0.01 > *P* > 0.001; ***: *P* < 0.001.

Figures 6A and 6B illustrate that both ON and OFF transient αRGCs in the MFA-treated group (ON transient αRGC = 4.10 × 10 Rh*/rod/second and OFF transient αRGC = 1.70 × 10³ Rh*/rod/second) exhibited higher light-evoked sensitivity compared to those in the Ringer’s solution group (ON transient αRGC = 9.12 × 10³ Rh*/rod/second and OFF transient αRGC = 1.62 × 10³ Rh*/rod/second). This effect was consistent across various full-field light sensitivity levels (2.4 × 10³, 1 × 10⁴, and 8.4 × 10⁴ Rh*rod^−1^s^−1^), indicating enhanced light responsiveness.

The light response curves showed a slight leftward shift in the MFA-injected group, as illustrated in Figures 6C and 6D. Specifically, the ON transient RGCs in Figure 6C shifted from 1.7 × 10³ in Ringer’s solution to 8.5 × 10² Rh*/rod seconds after MFA application, and the OFF transient RGCs in Figure 6D shifted from 5.0 × 10³ Rh*/rod seconds in Ringer’s solution to 3.98 × 10³ Rh*/rod seconds after MFA application.

Despite the trends of earlier spike initiation and a leftward shift in the light response curves, statistical analysis revealed no significant difference in light response thresholds between the MFA group (ON transient αRGCs = 3.23 ± 0.61, mean ± SEM, *n* = 4 and OFF transient αRGCs = 3.32 ± 0.17, *n* = 10) and the Ringer’s solution group (ON transient αRGCs = 3.92 ± 0.24, *n* = 4 and OFF transient αRGCs = 3.79 ± 0.22, *n* = 6) for both ON and OFF cells. This suggests that although MFA treatment may subtly enhance light responsiveness, it does not significantly lower the overall light response threshold under these experimental conditions.

### The Effect of MFA on EPSCs in ON αRGCs

We investigated whether the administration of MFA could suppress the reduction in input signals to RGCs by recording light-evoked EPSCs and IPSCs in two subtypes of αRGCs following corneal alkali burn injury.

**Figure 7.**
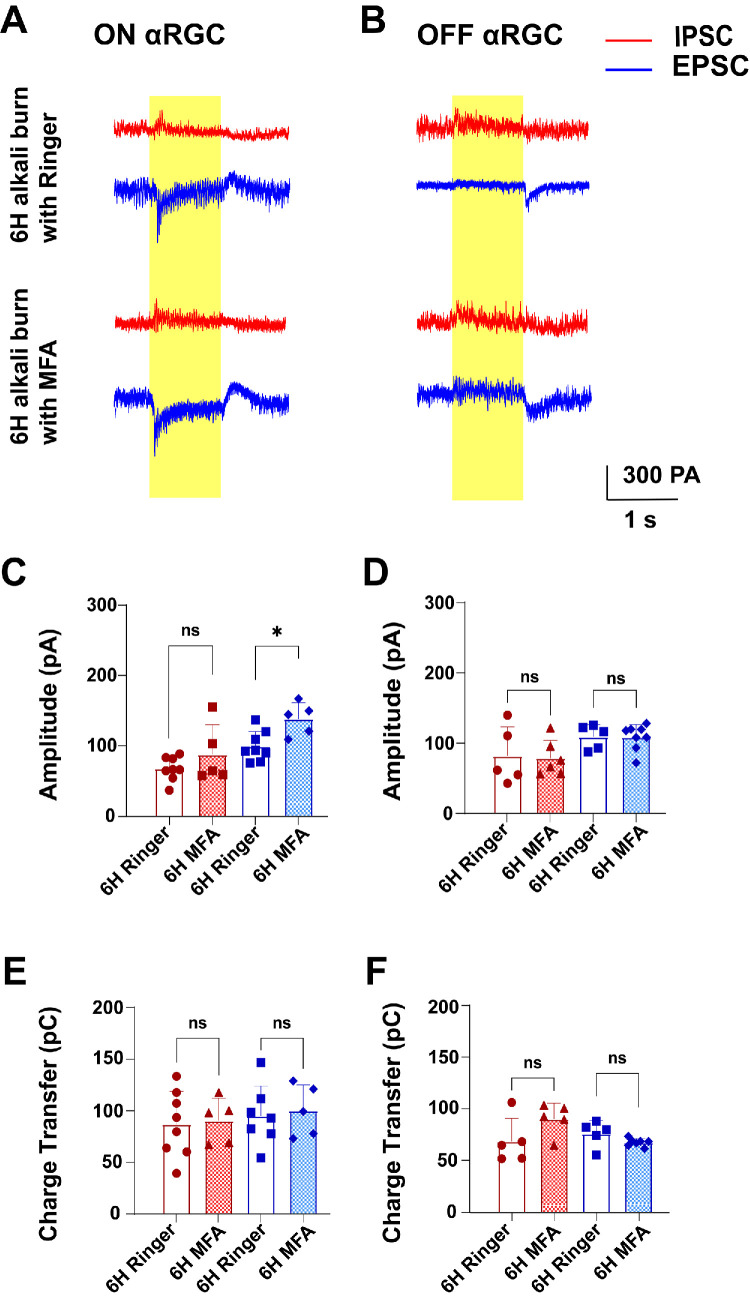
**Light-evoked EPECs and IPSCs in ON and OFF αRGCs of** **WT** **control and** **6-hour alkali burn groups.** EPSCs and IPSCs were recorded at holding potentials of −68 mV and +20 mV, respectively, with EPSCs shown in *blue* and IPSCs in *red*. (**A, B**) Images of light-evoked EPSCs and IPSCs in ON and OFF αRGCs from WT and 6-hour alkali burn mice. (**C, D**) Average peak amplitudes of light-evoked EPSCs and IPSCs in ON and OFF αRGCs. (**E, F**) Average total charge transferred by light-evoked EPSCs and IPSCs in ON and OFF αRGCs. ns, not significant, *P* > 0.05; *: *P* 0.05 > *P* > 0.01; **: *P* 0.01 > *P* > 0.001; ***: *P* < 0.001; ****: *P* < 0.0001.

Figure 7A shows representative EPSCs recorded at −68 mV and IPSCs at +20 mV under 1-second illumination at 525 nm from the same ON αRGC in each group. With MFA application, ON αRGCs exhibited notable changes in electrophysiological responses to full-field light stimulation, showing more pronounced inward currents in EPSCs (increasing from 136.7 ± 8.30 pA in Ringer’s solution to 149.5 ± 2.67 pA with MFA) and slightly larger outward currents in IPSCs (increasing from 70.8 ± 2.48 pA in Ringer’s solution to 101.3 ± 0.75 pA with MFA; Fig. 7A).

The administration of MFA significantly altered EPSCs of ON αRGCs, with a noticeable increase in peak amplitude under full-field light stimulation (Ringer = 109.70 ± 12.41 pA, mean ± SEM, *n* = 9 and MFA = 138.00 ± 10.40 pA, *n* = 5; unpaired *t*-test; see Fig. 7C). This suggests a modulatory effect on excitatory synaptic transmission. However, despite these changes, there was no statistically significant difference in the EPSC charge transfer (Fig. 7E; Ringer = 95.01 ± 10.97 pC, *n* = 7 and MFA = 100.00 ± 11.19 pC, *n* = 5; unpaired *t*-test), indicating stable overall synaptic charge. Regarding inhibitory currents, MFA increased the average peak amplitudes of IPSCs, but statistical analysis showed no significant difference between the MFA-treated group (87.74 ± 18.79 pA, *n* = 5) and the control group (67.48 ± 6.01 pA, *n* = 8; see Fig. 7C, Welch's *t*-test). Similarly, no significant difference was found in the IPSC charge transfer between the groups (Ringer = 86.91 ± 11.29 pC, *n* = 8 and MFA = 90.49 ± 9.75 pC, *n* = 5; see Fig. 7E, unpaired *t*-test).

We also examined whether MFA affected the input signals of OFF αRGCs by recording EPSCs and IPSCs (see Fig. 7B). The EPSC amplitudes were 128.2 ± 6.10 pA in Ringer’s solution and 128.1 ± 2.89 pA in MFA, whereas the IPSC amplitudes were 102.8 ± 7.75 pA in Ringer’s solution and 101.3 ± 2.96 pA in MFA (see Fig. 7B). Interestingly, the peak amplitudes (Ringer’s EPSC = 109.1 ± 7.79 pA, *n* = 5 and MFA EPSC = 108.4 ± 6.39 pA, *n* = 8; unpaired *t*-test; Ringer’s IPSC = 81.98 ± 18.49 pA, *n* = 5 and MFA IPSC = 78.70 ± 10.42 pA, *n* = 6; unpaired *t*-test; Fig. 7D) and charge transfer (Ringer’s EPSC = 75.75 ± 5.63 pC, *n* = 5 and MFA EPSC = 67.31 ± 1.39 pC, *n* = 7; Welch's *t*-test; Ringer’s IPSC = 68.45 ± 9.96 pC, *n* = 5 and MFA IPSC = 90.10 ± 6.87 pC, *n* = 5; unpaired *t*-test; Fig. 7F) of both inhibitory and excitatory currents in OFF αRGCs remained largely unchanged after MFA administration. No significant differences were observed between the MFA-treated and control groups.

## Discussion

Corneal alkali-induced trauma can lead to retinal damage and subsequent vision loss, potentially due to the apoptosis of RGCs. The study suggests that secondary cell death, mediated by the gap junction subunit Cx36, may play a crucial role in this process. Implementing a neuroprotective strategy that inhibits Cx36-mediated secondary cell death could be beneficial in the early stages following corneal alkali injury.

### Apoptosis of RGCs in Corneal Alkali Burn: A Six-Hour Post-Burn Model

Corneal alkali burns can induce apoptosis in various subtypes of αRGCs, starting in the far-peripheral retina and gradually spreading to the mid-peripheral and central regions A 6-hour post-injury time frame was chosen to examine the effects of alkali-induced corneal injury on retinal neurons, as it demonstrated a significant temporal and spatial progression of TUNEL-positive RGCs. Due to the eccentricity effect of RGC density in the mouse retina—where RGC density is highest in the central region and decreases toward the periphery—as well as the “snapshot” effect of assessing apoptosis at a specific time point (6 hours post-alkali burns), the mid-peripheral region may exhibit a statistically significant decrease in RGC apoptosis at this time, whereas the central and far-peripheral regions are less significantly affected (see Fig. 2). However, extending the observation period to 12 hours results in excessive apoptosis, complicating the study of biophysical properties.

The reduction in light sensitivity, particularly in transient αRGCs, may have important implications for visual processing and could contribute to visual disturbances following corneal injuries. These findings indicate that within the acute 6-hour period post-burn, both ON and OFF αRGCs experience significant impairment in light-evoked synaptic inhibition and excitation. This comprehensive alteration in retinal circuitry suggests a rapid and widespread impact of corneal injury on visual processing at the retinal level.

The observed changes in excitatory and inhibitory currents may contribute to the altered light sensitivity and visual disturbances associated with corneal alkali burns. Further research is necessary to elucidate the underlying mechanisms of these rapid functional changes and to explore potential therapeutic interventions that could mitigate the impact of corneal injuries on retinal function.

### Alkali Did not Play a Key Role in the Apoptosis of RGCs Following Corneal Alkali Burns

The mechanism of apoptosis in RGCs following corneal alkali burns involves a complex interaction of caspase activation and various apoptotic pathways. RGCs are particularly vulnerable to the damage, leading to their programmed cell death through caspase-dependent mechanisms.^29^

Alkali burns may induce RGC apoptosis through inflammatory responses and oxidative stress. These burns could cause an increase in TNF-α, which triggered RGC apoptosis via inflammatory pathways. This effect is intensified by the infiltration of peripheral monocytes and the activation of microglia, which release more TNF-α, further damaging the retina.^30^ Additionally, the activation of NADPH oxidases could boost the production of reactive oxygen species (ROS), intensifying oxidative stress and inflammation, both crucial in RGC apoptosis.^31^

To assess whether alkali base directly impacts the retina, we injected HEPES buffer into the vitreous cavity as a pH buffer to observe if it affected RGC apoptosis. The Ringer’s solution served as the vehicle control. Six hours after the corneal alkali burn, there was no significant difference in RGC apoptosis between the HEPES and Ringer’s control groups. This suggested that alkali may not be a primary factor in RGC death following corneal alkali burns. The vitreous body is substantial and effectively buffers sudden pH changes, providing protection to the eye.

### Microglial Activation and RGCs’ Apoptosis Following Corneal Alkali Burns

Apoptosis of RGCs following a corneal alkali burn may result from multiple mechanisms. To investigate potential immune-mediated contributions to RGC apoptosis, we examined the activation and quantitative changes in microglial cells post-burn (Supplementary Fig.). Microglia, the primary immune cells of the central nervous system, exhibited rapid and robust activation, signaling a significant immune response within the retina. This response may contribute to RGC apoptosis through the release of pro-inflammatory cytokines, ROS, or other neurotoxic factors.

The activation of microglia supports the notion that inflammatory responses, primarily mediated by retinal cytokines, play a crucial role in the pathophysiology of ocular alkali burns.^7^^,^^32^ This inflammatory component may complicate the pathogenesis of retinal damage following corneal alkali burns. There is a significant increase in microglial cells after an alkali burn, particularly in the far-peripheral area (rising from 43.48 ± 2.10 to 95.54 ± 3.87 cells/mm², *n* = 4), compared to the mid-peripheral (rising from 55.73 ± 1.17 to 96.77 ± 1.58 cell/mm², *n* = 4) and central areas (rising from 64.92 ± 2.12 to 105.30 ± 4.80 cell/mm², *n* = 4). Similar results were observed in Cx36 KO mice retinas, with a significant increase in microglial cells in the far-peripheral area (rising from 44.10 ± 3.61 to 80.23 ± 3.66 cell/mm², *n* = 4), compared to the mid-peripheral (rising from 50.83 ± 3.06 to 92.48 ± 5.14 cell/mm², *n* = 4) and central areas (rising from 52.67 ± 4.74 to 98.61 ± 5.14 cell/mm², *n* = 4; see Supplementary Fig.).

These findings suggest that the increased presence of microglial cells indicates that inflammatory responses play a crucial role in the pathophysiology of ocular alkali burns.

### Connexin 36 Played a Role in Acute Retinal Ganglion Cell Apoptosis Six Hours After a Corneal Alkali Burn

Our research reveals that the number of apoptotic cells in the retina increases progressively over time following a corneal alkali burn, with apoptosis advancing from the peripheral to the central retina and peaking at 24 hours. These findings were consistent with other animal models of alkali burns,^7^ indicating that corneal alkali burns triggered apoptosis of RGCs. A similar progressive pattern of RGC apoptosis was also observed in glaucoma. This similarity may help explain why glaucoma is a frequent complication in patients with ocular alkali burns.^33^

Electrical synapses, also known as gap junctions, modulate the visual signals and function as a regulator of the bidirectional flows of ions and small metabolites between neurons in the nervous system.^14^ Cx36 is the predominant subunit of gap junctions in the proximal mouse retina. Gap junction-mediated secondary cell death has been implicated in the loss of retinal neurons observed in various degenerative conditions, including retinitis pigmentosa, glaucoma, and ischemia.^34^^,^^35^ Cx36, a gap junction protein prevalent in the RGC layer, has been highlighted in recent studies for its role in mediating bystander cell death in the retina. Akopian et al.^11^ demonstrated that following cytochrome C injection into a single RGC, neighboring coupled RGCs exhibited TUNEL-positive reactions, suggesting gap junction-mediated secondary cell death. Furthermore, in excitotoxic models, Cx36 KO mice showed a 50% increase in cell survival compared with WT mice,^12^ indicating Cx36’s involvement in regulating secondary cell death.

**Figure 8.**
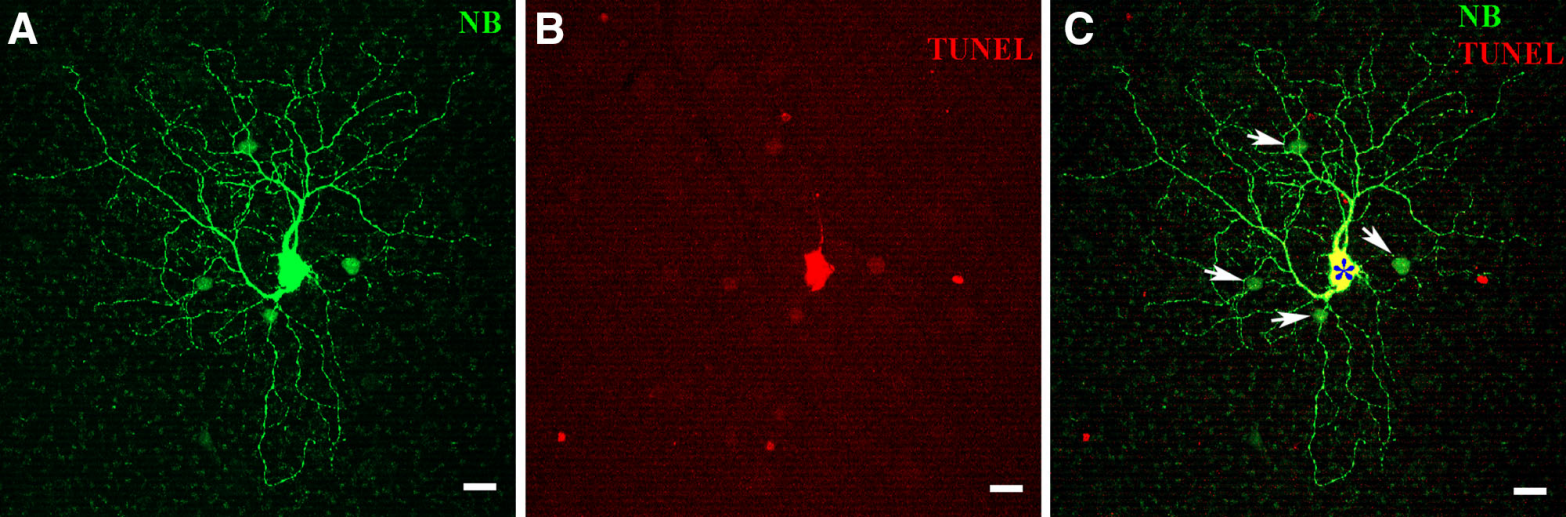
**Gap junction-mediated apoptosis in adjacent amacrine cells following CytC injection.** (**A**) This panel shows a representative confocal image of an ON αRGC along with its coupled amacrine cells in whole-mount retinas. The cells were injected with a mixture of Neurobiotin to visualize the gap junction coupling and CytC to induce apoptosis. (**B**) Coupled apoptotic amacrine cells are labeled using TUNEL staining. (**C**) The overlay of panels **A** and **B** demonstrates that apoptosis extends to neighboring amacrine cells coupled to the injected RGC (indicated by an *asterisk*). *Scale bars* represent 20 µm.

**Figure 9.**
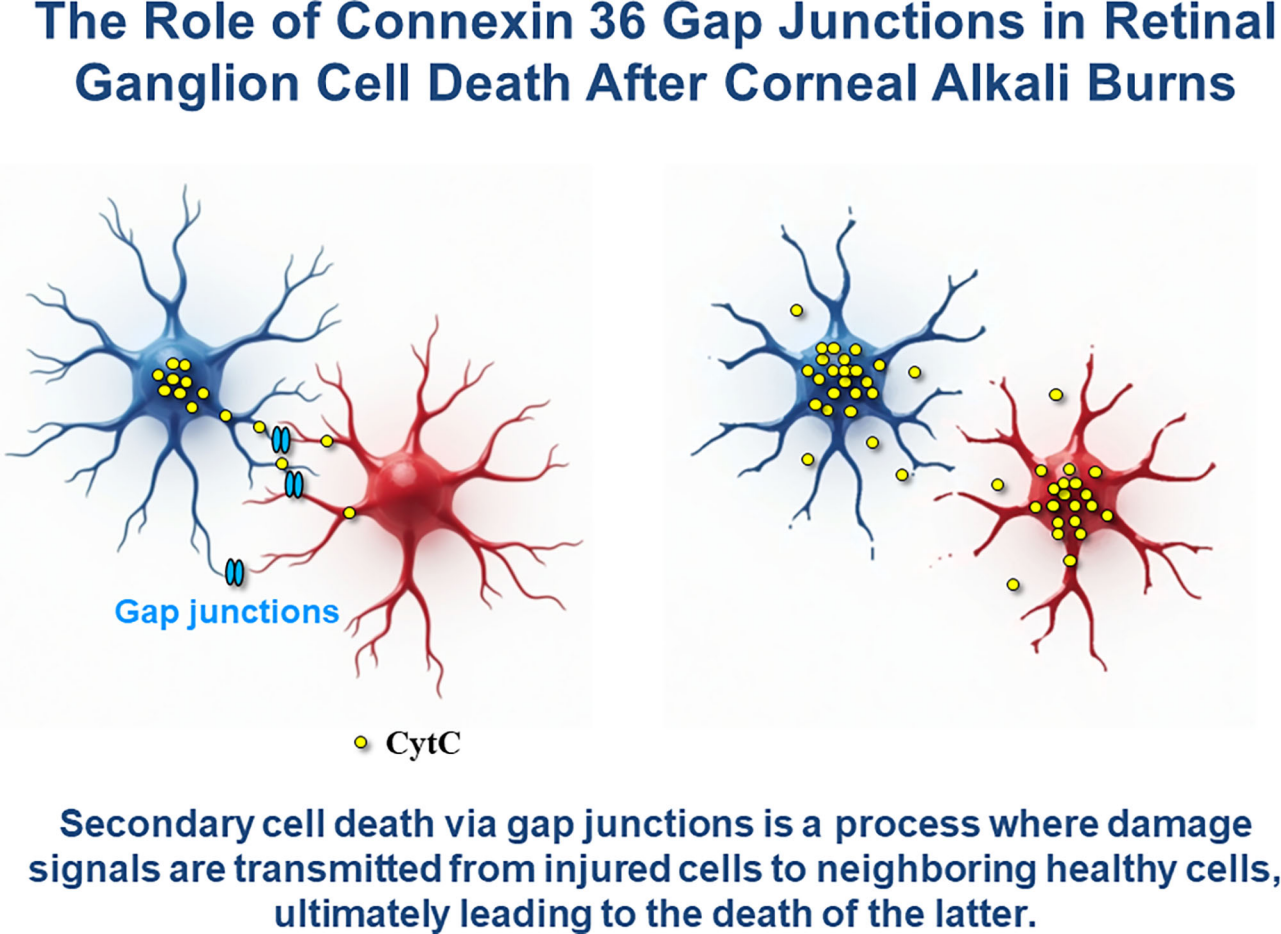
The graphic abstract summarizes secondary retinal ganglion cell death via connexin 36-related gap junctions following corneal alkali burns.

In our study, we replicated the experiment shown in Figure 8, focusing on ON αRGCs and their coupled amacrine cells in whole-mount retinas. ON αRGCs were injected with a mixture of Neurobiotin and CytC to visualize gap junction coupling with amacrine cells. The CytC injection induced apoptosis in the coupled amacrine cells, as confirmed by TUNEL staining. This experiment demonstrates that apoptosis can spread to neighboring amacrine cells through gap junctions with the injected RGC. A concentration of 10 mg/mL cytochrome C can induce secondary cell death in RGCs, whereas a concentration of 1 mg/mL is less effective. This observation suggests that the secondary death of retinal neurons may be dose-dependent (Fig. 9).

Six hours post-burn, both Cx36 KO mice and WT mice treated with MFA exhibited significantly reduced retinal apoptosis compared with controls. These findings underscore the role of Cx36 in mediating secondary cell death following corneal alkali burns. The protective effects observed with both genetic knockout and pharmacological inhibition of Cx36 not only corroborate previous findings but also enhance our understanding of Cx36’s involvement in corneal alkaline injuries. We propose that blocking Cx36 offers a neuroprotective effect on the retina after corneal alkali burns.

### Protecting RGCs From Corneal Alkali Burn With MFA Application

MFA, a gap junction antagonist, has demonstrated potential in protecting RGCs from damage and secondary cell death in experimental glaucoma.^12^ Already approved by the FDA for treating joint pain, muscular pain, and arthritis, MFA could play a crucial role in treating ocular alkali burns and preventing sight-threatening conditions if its neuroprotective effects were confirmed. Its existing approval could facilitate a faster regulatory pathway compared to a completely new chemical entity.

The study suggests that MFA may have a protective or restorative effect on RGC apoptosis. Although MFA did not significantly affect the light sensitivities of ON and OFF αRGCs, it did induce noticeable changes in both excitatory and inhibitory postsynaptic currents in ON αRGCs, with minimal impact on the synaptic inputs of OFF αRGCs.

We selected the 3-day time point to observe RGC survival after alkali exposures. The surviving RGCs, defined as Brn3a-positive and TUNEL-negative cells, were quantified as follows. In the WT group at 3 days post-injury, the densities were 137 ± 20 cells/mm² in the far-peripheral retina, 852 ± 140 cells/mm² in the mid-peripheral retina, and 1430 ± 83 cells/mm² in the central retina (*n* = 4). In Cx36 KO mice, the corresponding values were 966 ± 379, 1856 ± 355, and 2790 ± 211 cells/mm² (*n* = 4), respectively. Similarly, in the MFA-treated group, the densities were 765 ± 214, 1774 ± 115, and 2734 ± 77 cells/mm² in the far-peripheral, mid-peripheral, and central retina (*n* = 4), respectively. The results indicated that both genetic deletion of Cx36 and pharmacological blockade may provide neuroprotection to RGCs after alkali injury.

The phases of alkali burn ocular injury are typically classified as acute (0–7 days), early reparative (7–21 days), and late reparative (beyond 21 days). The prognosis for ocular alkali burns depends on several factors, including the severity of the burn, time to treatment, post-immune-related injury, and other unknown mechanisms, making the process more complex than we originally anticipated. Nevertheless, early and aggressive intervention is generally believed to minimize damage and improve the likelihood of a favorable outcome.

These findings could potentially identify new targets for therapeutic intervention in RGC injury and provide evidence for the efficacy of MFA in treating alkali burns, bringing its possible clinical application one step closer.

## Supplementary Material

Supplement 1
