# Supplementary material for: The Role of Connexin 36 Gap Junctions in Retinal Ganglion Cell Death After Corneal Alkali Burns
Source: Invest Ophthalmol Vis Sci. 2025 Sep 18;66(12):43. doi: 10.1167/iovs.66.12.43 (PMC12449816; doi:10.1167/iovs.66.12.43)
Supplement: Supplement 1 [file iovs-66-12-43_s001.docx]

**Supplementary Material**


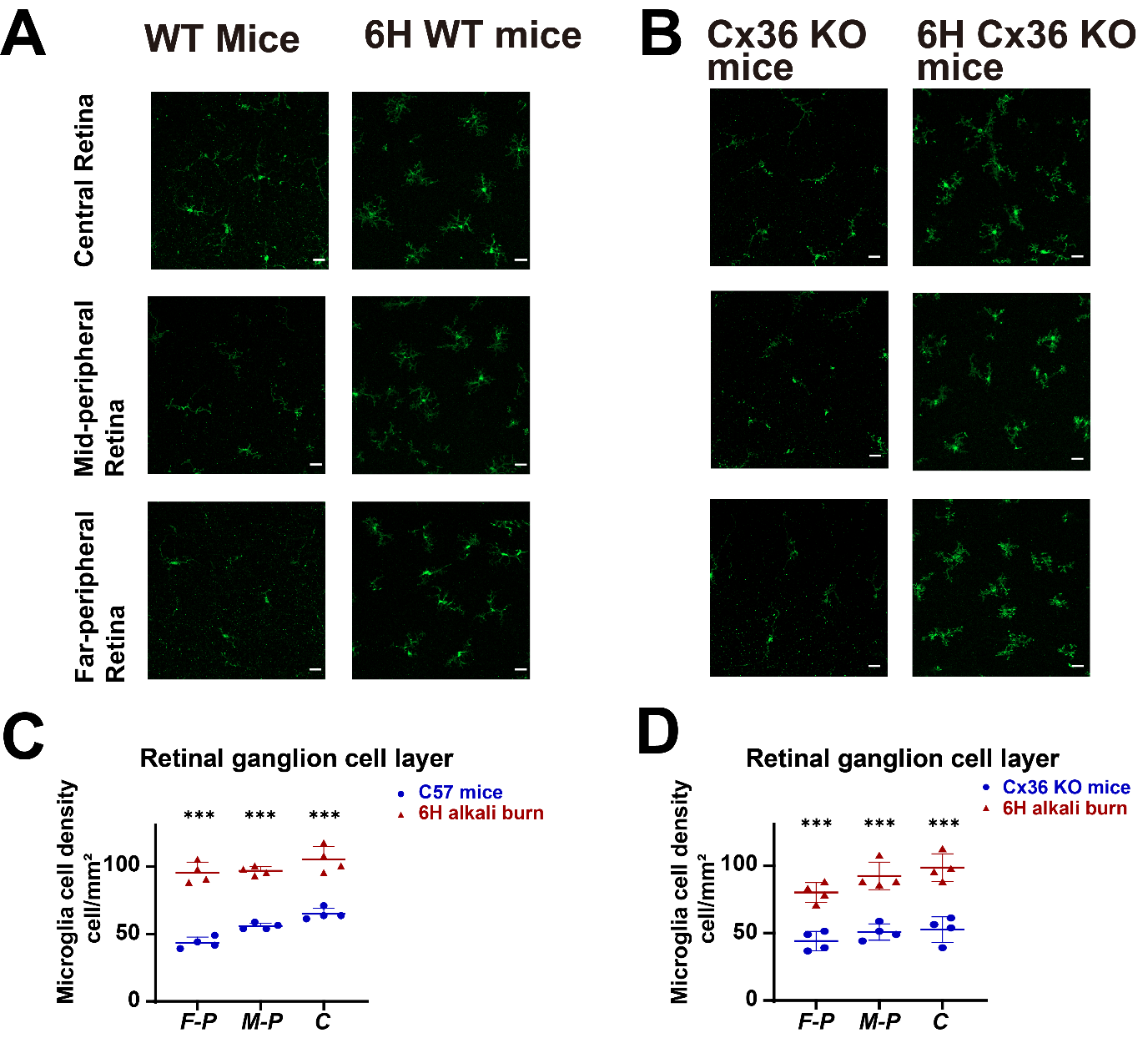


**Supplementary Figure: Microglial changes following a six-hour corneal alkali burn.**

(A) Confocal images illustrating the far-peripheral (F-P), mid-peripheral (M-P), and central (C) regions of the retinas in wild-type (WT) and six-hours after corneal alkali burn.

(B) Confocal images of the far-peripheral, mid-peripheral, and central retinas in Cx36 KO HOMO mice, both untreated and six-hour post-burn. Microglia were visualized using anti-Iba1 immunolabeling (green).

(C) Quantitative analysis of microglial counted per image in WT mice, comparing untreated and post-burn conditions.

(D) Quantitative analysis of microglial counted per image in Cx36 KO HOMO mice, comparing untreated and post-burn conditions.

(ns: p > 0.05; *: 0.05 > p > 0.01; **: 0.01 > p > 0.001; ***: p < 0.001)
